# Gastroduodenal artery aneurysm, diagnosis, clinical presentation and management: a concise review

**DOI:** 10.1186/1750-1164-7-4

**Published:** 2013-04-16

**Authors:** Nicholas Habib, Samer Hassan, Rafik Abdou, Estelle Torbey, Homam Alkaied, Theodore Maniatis, Basem Azab, Michel Chalhoub, Kassem Harris

**Affiliations:** 1 Staten Island University Hospital, 475 Seaview ave, Staten Island, NY, 10305, USA

## Abstract

Gastroduodenal artery (GDA) aneurysms are rare but a potentially fatal condition if rupture occurs. They represent about 1.5% of all visceral artery (VAA) aneurysms and are divided into true and pseudoaneurysms depending on the etiologic factors underlying their development. Atherosclerosis and pancreatitis are the two most common risk factors. Making the diagnosis can be complex and often requires the use of Computed Tomography and angiography. The later adds the advantage of being a therapeutic option to prevent or stop bleeding. If this fails, surgery is still regarded as the standard for accomplishing a definite treatment.

## Introduction

Visceral artery aneurysms (VAA) are infrequent conditions characterized by a wide range of clinical presentations and various clinical outcomes. Depending on the mechanism of formation and etiologic factors, they can be divided into true aneurysms or pseudoaneurysms. True aneurysms are the results of vessel wall abnormalities while pseudoaneurysms occur after vascular injuries or erosions such as in trauma or inflammation [1], (i.e. pancreatitis, autoimmune disorders, vascular intervention, laparoscopic cholecystectomy and hepatic transplantation [2].

## Epidemiology

Pseudoaneurysms are mostly a condition of the middle age and are most commonly found between 50 and 58 years of age [3,4]. The male/female ratio is 4.5:1 and the mean size 3.6 cm [3].

They have been reported in almost all the visceral arteries [5] but are most commonly seen in the splenic artery (46%), followed by the renal artery (22%), the hepatic artery (16.2%) and the pancreaticoduodenal artery (1.3%) [6]. Those involving the gastroduodenal artery comprise only 1.5% of all reported VAA [7] and most of them are pseudoaneurysms due to their high occurrence rates in the setting of pancreatitis. Thus, true aneurysms in the pancreaticoduodeal and gastroduodenal arteries are extremely rare and represent only 3.5% of all VAA [5]. In a review of the English literature over a 25 year period from 1970 to 1995, pancreatitis was found to be the most common associated condition with gastroduodenal artery aneurysm accounting for 47% of all cases followed by ethanol abuse (25%), peptic ulcer disease (17%) and cholecystectomy (3%) [5,7,8]. Other reported causes include congenital abnormalities (such as Marfan syndrome and Ehlers- Danlos syndrome [9]), liver cirrhosis [10], other vascular abnormalities such as fibro-muscular dysplasia, polyarteritis nodosa and predisposing events such trauma and septic emboli [11].

## Pathophysiology

The pathogenesis of GDA aneurysms is not fully understood. Trauma, hypertension and atherosclerosis have been cited as potential risk factors for true aneurysms [12]. The pathophysiologic changes that underlay the development of true gastroduodenal artery aneurysms comprise mainly atherosclerosis of the celiac artery with subsequent stenosis but also rarely congenital absence of the celiac axis [9]. These two circumstances can be distinguished by the morphology of the collaterals that develop. The collateral vessels that form early on have usually parallel walls, are of uniform caliber and are limited to one or two vessels. In contrary, arteriosclerotic collaterals are more abundant, dilated and tortuous and hence more prone to aneursymal formation within the vessel’s wall. This occurs regardless of the location of the stenosis [13].

The pancreaticoduodenal artery is the main collateral pathway between the celiac axis and the superior mesenteric artery. Increased blood flow in the pancreaticoduodenal artery, as compensation for celiac artery stenosis, may cause a pancreaticoduodenal artery aneurysm [14]. The same theory suggests that occlusion or stenosis of the superior mesenteric artery or celiac axis could be an etiologic factor predisposing to the formation of a gastroduodenal artery aneurysm [13,15,16].

As for pseudoaneurysms, inflammation with the most common cause being pancreatitis, results in vascular wall destruction that is mediated by pancreatic proteolytic enzymes leading to pseudoaneurysm formation [17].

### Clinical presentation

After reviewing the literature extending from1956 to 2011, 74 cases describing GDA aneurysms were collected from the Japanese and English literature. A gastrointestinal hemorrhage secondary to rupture of the aneurysm was found to be the most common clinical presentation (52%) while only 7.5% of GDA aneurysms remained asymptomatic (Table 1). Abdominal pain is the second most common symptom and occurs in 46% of cases. The mortality rate with rupture is about 40% [18] and depends on the severity, speed of the blood loss and the anatomical site of the rupture. The highest mortality rate comes from rupture into the duodenum approaching 21% [8,19,20]. These patients present with hematemesis, melena, and hemodynamic shock [21]. Less frequently, patients with GDA can present with retroperitoneal or intra-peritoneal bleeds [19,22-24] with a 19% mortality rate. This could lead to gastric outlet obstruction [22] and other nonspecific symptoms such as vomiting, diarrhea and jaundice secondary to compressive hematoma or external pressure by the aneurysm [19,25,26]. In addition, bleeding into the pancreatic duct manifesting as recurrent episodes of hemosuccus pancreaticus have been reported as well as bleeding into the common bile duct [27]. The presence of a pulsatile abdominal mass with or without a bruit on auscultation could be the sole warning sign [22] and should raise the suspicion of a GDA aneurysm with prompt diagnostic work up to preclude the worst outcome.

**Table 1:** Common presenting symptoms of GDA

	
1. Rupture (hematemesis, melena, shock)	
2. Abdominal pain	
3. Gastric outlet obstruction	
4. Compressive symptoms (nausea, vomiting)	
5. Hemobilia/ Hemosuccus pancreaticus	
6. Pulsatile abdominal mass/ Bruit	
7. Asymptomatic	

### Diagnosis

Prior to the era of sophisticated imaging modalities the majority of cases of GDA aneurysms were undiagnosed until rupture occurred. Currently with the various imaging studies available, an increasingly larger number of cases are being incidentally detected in asymptomatic patients.

**Figure 1:**
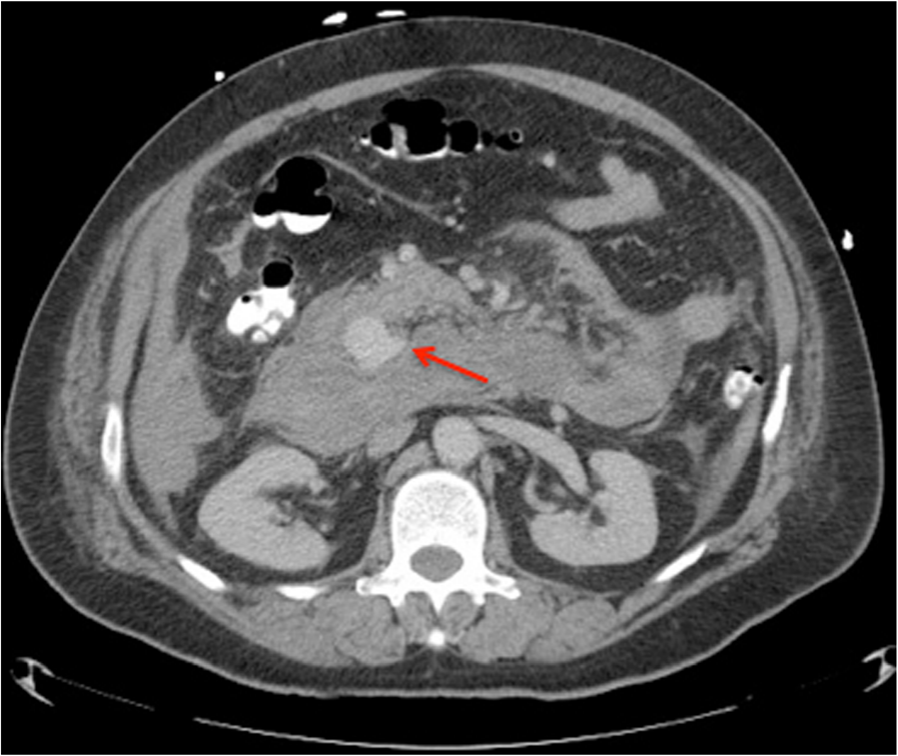
Abdominal contrast enhanced computed tomography reveals retroperitoneal aneurysm (arrow) that is suspected to be arising from the gastroduodenal artery or one of its branches.

The gold standard diagnostic test is visceral angiography [28] and it serves both diagnostic and therapeutic purposes by delineating the arterial anatomy and allowing therapeutic intervention [28,29]. It has the highest sensitivity (100%) followed by computed tomography (CT) (67%) and ultrasonography (US) (50%). CT scan has the advantage of being non invasive and localizing the aneurysm with its relations to surrounding structures (Figure 1). When performed in a patient with pancreatitis, CT scan can reveal a homogeneously enhancing structure within or adjacent to a pseudocyst which is highly suggestive of an associated pseudoaneurysm [30]. Three-dimensional CT adds to the accuracy of the study [4].

Doppler US may reveal turbulent arterial blood flow within or adjacent to a pseudocyst which is also suspicious for an aneurysm.

Plain X-ray of the abdomen is a rarely helpful study but may show shell-like calcifications in an atherosclerotic aneurysm [6].

New modalities such as Contrast-enhanced 3-dimensional magnetic resonance angiography or multi-detector row computed tomography have been reported to be as effective as visceral angiography in the diagnosis of abdominal vascular lesions [31,32]. Other diagnostic studies are available including Pulse Doppler US, color Doppler US, endoscopic ultrasound and magnetic resonance imaging [33-35] but are less frequently used.

## Treatment

**Figure 2:**
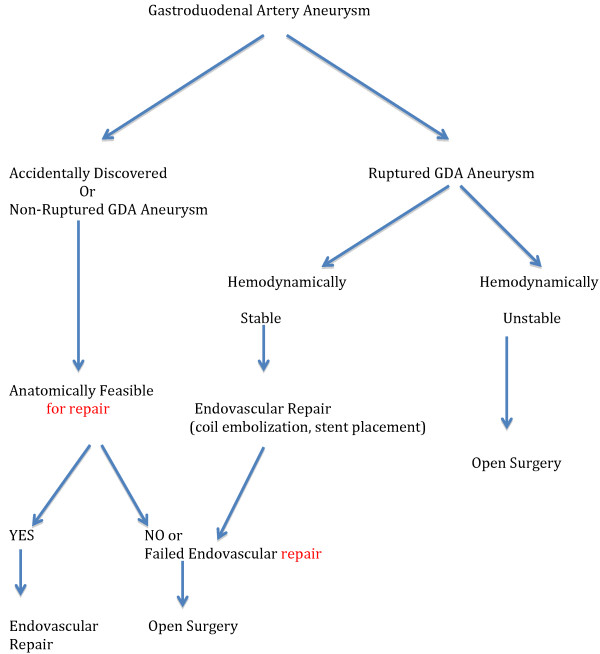
Treatment algorithm of GDA aneurysms.

**Figure 3:**
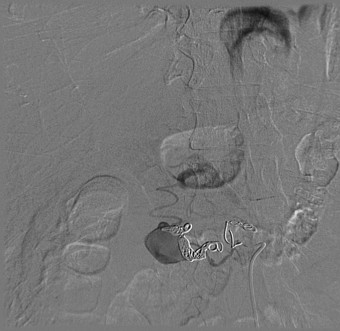
Post embolization angiography showing insignificant residual filling of the gastroduodenal artery aneurysm.

Once a GDA aneurysm ruptures, the patient faces a life threatening condition that could rapidly lead to death in 40% of cases [18]. Therefore, it is of utmost importance to diagnose and treat GDA aneurysms before a fatal complication occurs. Such a complication is not always related to the size of the aneurysm and therefore treatment should be planed as soon as a diagnosis is made [10]. Therapeutic strategies include surgical (revascularization, vessel ligature, aneurysmal sac exclusion) or endovascular interventions (coil embolization, stent placement), (Figure 2). The choice of the therapeutic procedure is made on individual basis and depends on the presenting symptom, the location of the aneurysm, and general condition of the patient and the risk of organ ischemia after the intervention [10,15]. The traditional therapy of visceral artery aneurysms has been the surgical resection or ligation of the aneurysm. Recently, endovascular treatment, such as trans-catheter embolization, has been an alternative to open surgical repair and has become increasing popular [16]. Open surgical approach of patients suffering from a VAA is a safe and life-saving procedure. In 88.2% of patients, the treatment remained successful after a mean follow-up of 54 months [36]. Moreover, emergent surgery is the treatment of choice in case of aneurysmal rupture in a hemodynamically unstable patient and consists of ligation, aneurysmorrhaphy or bypass surgery [2]. If the condition of the patient allows it, the less invasive endovascular options should be exhausted before proceeding with surgery. If bleeding recurs or cannot be controlled, vascular surgery will still be a feasible alternative [37]. Vascular reconstruction after exclusion of the aneurysm is not always necessary, as collaterals almost always exist between the visceral arteries. For example, vascular supply to the stomach comes from the both the GDA and the Superior Mesenteric Artery (SMA). Hence, vascular reconstruction is not essential after resection of a GDA aneurysm unless there is celiac artery occlusion, as ligation of the GDA may cause gangrene of the gallbladder and stomach, splenic necrosis or other disastrous consequences [1,15,38]. However, adequate collateral flow should be documented with preoperative imaging if permitted. CT and magnetic resonance angiography demonstrate excellent resolution for preoperative planning but angiography may allow better evaluation of real time flow dynamics [31]. For those patients with celiac artery or SMA stenosis, trans-luminal angioplasty would be one way of avoiding the risk of organ ischemia before or after surgical resection of the aneurysm [39]. In cases of erosion into the surrounding bowel structure, endoscopic techniques to identify and stop the source of bleed can be attempted initially as long as the patient’s condition allows it [15]. Therefore, the endovascular option being a less aggressive approach, performed under local anesthesia offers a good therapeutic alternative for those patients who are unfit for surgical treatment due to incapacitating comorbidities [40] and is associated with a shorter hospital stay (Figure 3).

Endovascular options include embolization of the aneurysms or stent graft deployment [41,42] (Figure 3). Some anatomical conditions are required though for technical feasibility of these procedures (saccular aneurysm with a narrow neck, fusiform aneurysm with adequate collateral flow, aneurysm of a vessel supplying an organ that has multiple arterial sources) [41,43].

Even though this less aggressive option plays an important role in high risk surgical candidates, it has its potential complications such as visceral ischemia resulting in sacrifice of the involved visceral vessel, end-organ thrombosis, and late-term vessel recanalization. Transcatheter embolization is the most popular endovascular intervention performed despite the potential risk of visceral ischemia and organ infarction [44]. Other complications include coil/stent migration [45,46], intra-procedural aneurysm dissection, or rupture [47], embolisms, access artery pseudoaneurysms and contrast-induced nephropathy. In contrast, surgical interventions have their own share of complications such as paralytic ileus, wound infection, massive bleeding, or acute pancreatitis [41]. These complications have a significantly higher rate of occurrence in patients with previous abdominal surgery where adhesions are present, making the endovascular approach the preferred treatment option in those patients [38].

Despite the fact that endovascular treatments do not represent a standard option and require both a specific training and a learning curve, the development of new technologies, such as the multilayer stent, could offer a new alternative to VAA treatment, particularly in high-risk patients [47].

## Conclusions

In conclusion, physicians might only encounter GDA aneurysms as an incidental finding on CT scans. In unfortunate patients, rupture might occur and lead to a fatal outcome if an emergent intervention is not made. Depending on the patient’s condition, the decision to proceed with angiography or surgery should be taken without any delay to prevent the worst outcome.

## Competing interests

The authors declare that they have no competing interests.

## Authors’ contributions

KH contributed to developing the review concept. KH, NH, SH, RA, and ET participated in writing the manuscript, interpreting the gathered data and approving the manuscript for submission. HA, TM, MC, and BA participated in research and data gathering. All authors read and approved the final manuscript.
